# Proximal tubular epithelia-specific transcriptomics of diabetic mice treated with dapagliflozin

**DOI:** 10.1016/j.heliyon.2022.e10615

**Published:** 2022-09-13

**Authors:** Noriko Uehara-Watanabe, Natsuko Okuno-Ozeki, Itaru Nakamura, Tomohiro Nakata, Kunihiro Nakai, Aya Yagi-Tomita, Tomoharu Ida, Noriyuki Yamashita, Michitsugu Kamezaki, Yuhei Kirita, Satoaki Matoba, Keiichi Tamagaki, Tetsuro Kusaba

**Affiliations:** a Department of Nephrology, Graduate School of Medical Science, Kyoto Prefectural University of Medicine, Kyoto, Japan; b Department of Cardiovascular Medicine, Graduate School of Medical Science, Kyoto Prefectural University of Medicine, Kyoto, Japan

**Keywords:** Diabetic nephropathy, Proximal tubular epithelial cell, Dapagliflozin, Hypoxia

## Abstract

Based on recent clinical trials using sodium-glucose co-transporter 2 inhibitor (SGLT2i) demonstrating the significant improvement of outcomes of diabetic kidney disease (DKD), the paradigm shift from “glomerulocentric” to “tubule centric” pathophysiology in DKD progression has been highlighted. Several responsible mechanisms for renoprotective effects by SGLT2i have been proposed recently, but the changes in proximal tubule-specific gene expression by SGLT2i in diabetic mice have not been elucidated. We report the analysis of the proximal tubular-specific pathway, demonstrating the downregulation of oxidative phosphorylation in dapagliflozin-treated *db/db* mice, a type 2 diabetic model. After 8-week treatment of dapagliflozin for *db/db* mice having a proximal tubule-specific tdTomato reporter, tdTomato-positive cells were isolated by FACS. Pathway analysis of RNA sequencing of isolated tubular epithelia revealed that oxidative phosphorylation was downregulated in dapagliflozin-treated mice. However, depletion of renal tissue ATP content in *db/db* mice was ameliorated by dapagliflozin administration. Pimonidazole staining demonstrated renal cortical tissue hypoxia in *db/db* mice, which was improved by dapagliflozin administration. This study suggests that dapagliflozin can ameliorate the excessive oxygen and ATP consumption, and subsequent tissue hypoxia in the diabetic kidney, which may explain, in part, the responsible mechanisms of the renoprotective effects of dapagliflozin.

## Highlights

• Oxidative phosphorylation is downregulated by dapagliflozin in the proximal tubules of diabetic kidneys.
• Renal tissue ATP content increased in dapagliflozin-treated diabetic mice.
• Dapagliflozin improved renal cortical hypoxia in type 2 diabetic mice.

## 1 Introduction

Recent clinical trials using sodium-glucose transporter (SGLT)2 demonstrated a notable risk reduction for renal outcomes, including decreases in eGFR or ESRD, in DKD patients [1, 2, 3]. Furthermore, more recent clinical trials using canagliflozin and dapagliflozin (Dapa) demonstrated the efficacy of these drugs for renal outcomes even in DKD patients with reduced kidney function [4, 5]. The molecular mechanisms of the renoprotective effects of SGLT2 inhibitors have been investigated in clinical and basic studies, and are thought to be attributed not only to their blood glucose reducing effects, but also to their effects on sodium excretion [6]. We recently demonstrated the renoprotection by ipragliflozin through 3 major mechanisms, i.e. reduction of oxidative stress in both tubular epithelia and glomerular podocytes, normalization of glomerular hyperfiltration, and amelioration of renal tissue hypoxia [7].

Considering the improvement of renal prognosis by SGLT2 inhibition in DKD patients, the paradigm shift from “glomerulocentric” to “tubule centric” has emerged in the pathophysiology of DKD progression [8, 9]. Although several renoprotective mechanisms by SGLT2 inhibitors have been reported, proximal tubule-specific analysis of detailed molecular mechanisms is limited. Several investigations *in vitro* have been performed, but one concern in *in vitro* experiments is the loss of this polarity and subsequent loss of expression of membrane transporters, including SGLT2, in tubular epithelia under culture conditions [10, 11]. In order to overcome these limitations, we performed transcriptomics analysis of proximal tubular epithelia sorted by flow cytometry from type 2 diabetic mice with a proximal tubule-specific fluorescence reporter. We confirmed the effects of dapagliflozin specifically in the proximal tubular epithelia under diabetic conditions.

## 2 Materials and methods

### 2.1 Mice and animal experiments

In order to generate type 2 diabetic mice having the tdTomato reporter specifically expressed in proximal tubular epithelia, we have recently generated mice with the CreERT2 cassette in the SLC34a1 locus (SLC34a1GCE), which can induce the expression of the Cre recombinase specifically in the proximal tubules after tamoxifen administration [12]. SLC34a1GCE mice were crossed with R26tdTomato mice, in which tdTomato fluorescence reporter is expressed after Cre-mediated excision of the floxed stop cassette. After further crossing with BL6 m+/+Leprdb (*db/m*) (CLEA Japan, Inc., Tokyo, Japan), we finally obtained type 2 diabetic (*db/db*) mice and non-diabetic heterozygote (*db/m*) mice, which have SLC34a1GCE and R26tdTomato transgenes. For genetic labeling of proximal tubules, tamoxifen (Sigma-Aldrich Co., LCC., St. Louis, MO) was dissolved in 3% (vol/vol) ethanol containing corn oil (Sigma Aldrich). Tamoxifen (3 mg/mouse) was injected intraperitoneally every other day 5 times into 8-week-old mice. For SGLT2 inhibition *in vivo*, 1.5 mg/kg of dapagliflozin (AstraZeneca Cambridge, UK) was administered through drinking water every day for 8 weeks as described previously [13]. Body weights and blood glucose levels were measured at 17:00 after 8 h fasting every 2 weeks. Blood glucose levels were measured by a glucometer (Glutest Every, Sanwa Kagaku Kenkyusho Co., Ltd., Aichi, Japan). After the experimental period, mice were anesthetized with isoflurane and euthanized. All experiments were approved by the Experimental Animals Committees of Kyoto Prefectural University of Medicine, and were performed in accordance with the institutional guidelines and Guidelines for Proper Conduct of Animal Experiments by the Science Council of Japan.

### 2.2 Tissue preparation and histological analysis

For frozen sections, kidneys were fixed with ice-cold 4% paraformaldehyde (Wako Pure Chemical Industries, Ltd., Osaka, Japan) for 1 h, incubated in 30% (vol/vol) sucrose in PBS at 4 °C for 16 h, embedded in optimum cutting temperature compound (Sakura FineTek Japan co., Ltd., Tokyo, Japan). For paraffin sections, the kidneys were fixed with 4% paraformaldehyde and embedded in paraffin by Applied Medical Research Laboratory (Osaka, Japan).

Kidney histology was examined on formalin sections stained with PAS. The area of approximately 100 glomeruli was measured using a BZ-X700/BZ-X710 microscope (Keyence Corporation, Osaka, Japan), and the average area was calculated as previously described [7].

### 2.3 Immunohistochemistry and assessment of renal tissue hypoxia

Renal tissue hypoxia was visualized by immunostaining of pimonidazole as described previously [7]. One hour before sacrifice, pimonidazole (HypoxyprobeTM-1, Natural Pharmacia International, Inc., Burlington, MA) in saline was injected intraperitoneally at a dose of 60 mg/kg. After deparaffinization and antigen retrieval, endogenous peroxidase was quenched with 3.0% hydrogen peroxide in methanol. Blocking was performed using 3.0% BSA in PBS. Then, sections were incubated with anti-pimonidazole antibody (PAb2627, 1:500, Natural Pharmacia International, Burlington, MA) or anti-Tom20 antibody (sc-11415, 1:250, Santa Cruz Biotechnology, Dallas, TX), followed by an incubation with HRP-conjugated secondary antibodies (Abcam, ab80437). Thereafter, sections were incubated with diaminobenzidine chromogenic substrate (K3468, Agilent Technologies, Inc., Santa Clara, CA), followed by counterstaining with hematoxylin. All sections were observed using an Eclipse E600 microscope (Nikon Corporation, Tokyo, Japan). Renal hypoxia was semi-quantitated by the immunostaining of pimonidazole as described previously [7]: the proportion of pimonidazole-positive renal tubules in the cortex was calculated by dividing the number of pimonidazole-positive renal tubules by the total number of renal tubules in each field. Hypoxia in the renal medulla was scored according to the following intensities: 0, negative; 1, weak; 2, moderate; and 3, strong staining.

### 2.4 Immunofluorescence analysis

For immunofluorescence, sections were rehydrated and permeabilized with 0.5% Triton X-100 in PBS for 5 min. Samples were blocked with 5% of normal goat serum in PBS and incubated with FITC-conjugated anti-LTL (Vector Labs, Cat. # FL-1321, 1:1000). Nuclear counterstaining was performed using DAPI or DRAQ5 (DR50050; BioStatus, Leicestershire, UK; 1:2000), followed by mounting in Prolong-Gold (Thermo Fisher Scientific). Images were obtained by confocal microscopy (FV1000; Olympus, Tokyo, Japan). tdTomato + tubules among LTL + tubules were quantified from 5 of 25 consecutive non-overlapping cortical fields in each kidney under high magnification (n = 5).

### 2.5 Separation of tdTomato-positive proximal tubular epithelia using FACS

tdTomato + labeled proximal tubular epithelia was isolated by FACS as described previously [14]. In detail, after mincing the kidney cortex, a single-cell suspension was generated via Liberase TL (5401020001: Sigma-Aldrich) and 60 units/ml of DNAse Inhibitor (D5025: Sigma-Aldrich) for 30 min at 37 °C. after washing twice with PBS, cells were filtered through 70- and 40-μm cell strainers, resuspended in PBS and 2% FBS with 1000:1 DAPI (1 mg/mL), and subjected to FACS using SH800 (SONY). During FACS, DAPI + dead cells were excluded. TdTomato + DAPI- cells were collected in DMEM and 10% FBS.

### 2.6 Measurement of ATP levels in renal tissues

ATP concentrations in renal cortex samples were measured using an ATP-bioluminescence assay with the “Tissue” ATP assay Kit (TOYO B-Net Co. Ltd, Tokyo, Japan) according to the manufacturer’s instructions. Relative ATP concentrations to tissue volume are expressed as the ratio to that of *db/m* mice kidneys.

### 2.7 RNA extraction and RNA sequencing

Total RNA was isolated from FACS-sorted cells from 3 mice in each experimental group using TRIzol (Life Technologies, Inc., Carlsbad, CA) and Direct-zol™ RNA MiniPrep (Zymo Research Corporation., Irvine, CA). Subsequently, RNA samples were provided to the NGS core facility of the Genome Information Research Center at the Research Institute for Microbial Diseases of Osaka University for library construction and sequencing. The library preparation was performed using a TruSeq stranded mRNA sample prep kit (Illumina, San Diego, CA) according to the manufacturer’s instructions. Sequencing was performed on an Illumina HiSeq 2500 platform in a 101-bp single-end mode. Sequenced reads were mapped to the mouse reference genome sequences (mm10) using TopHat v 2.0.13 (https://ccb.jhu.edu/software/tophat/manual.shtml) in combination with Bowtie2 ver. 2.2.3 (http://bowtie-bio.sourceforge.net/bowtie2/manual.shtml) and SAMtools ver. 0.1.19 (http://www.htslib.org/doc/#publications). The fragments per kilobase of exon per million mapped fragments (FPKMs) was calculated using Cufflinks version 2.2.1 (http://cole-trapnell-lab.github.io/cufflinks/) [15].

### 2.8 Bioinformatic analysis

Data analysis was carried out using R software. Genes with fewer than 0.3 FPKM in one-sixth of the samples were excluded from further analysis. edgeR package was used for differential expression analysis [16]. Genes with FDR <0.1 and absolute log2 fold change >1 were considered significantly differentially expressed genes. Performance of the samples was assessed with principal component analysis (PCA) plots. Kyoto Encyclopedia of Genes and Genomes (KEGG) pathway analysis (https://www.genome.jp/kegg/kegg1.html) was performed using iDEP version 0.93 (http://bioinformatics.sdstate.edu/idep93/) with the generally applicable gene-set enrichment for pathway analysis (GAGE) method [17].

### 2.9 Statistics

Results are expressed as the mean ± SEM. Each experiment was performed using at least 5 mice per group. Statistical analysis was performed by analysis of variance and Tukey’s post hoc test for comparison of multiple variables. P-values < 0.05 were considered significant.

## 3 Results

### 3.1 Physiological and histological analyses of dapagliflozin-treated type 2 diabetic mice having a proximal tubular reporter

**Figure 1:**
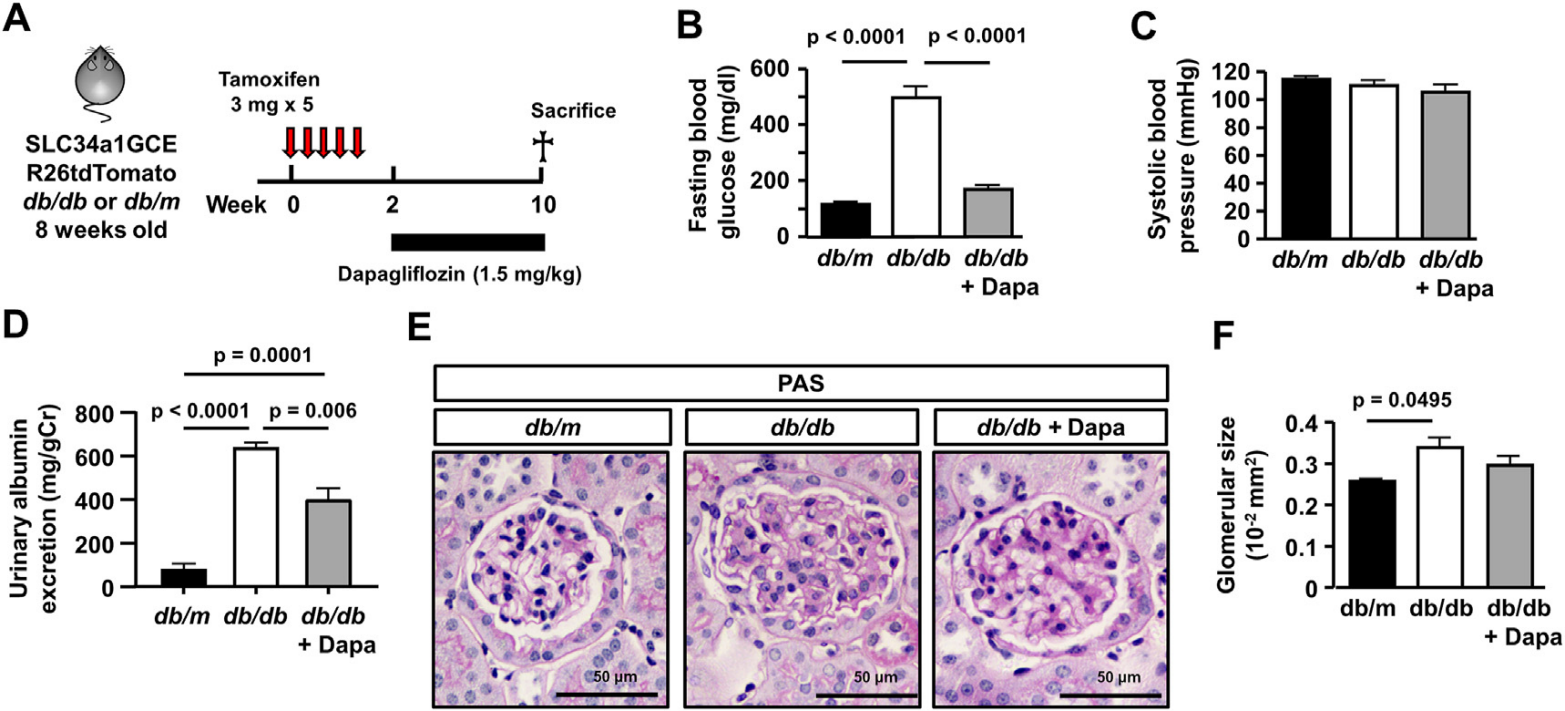
**Physiological and histological analyses of dapagliflozin-treated type 2 diabetic mice.** (A) Experimental protocol. Five injections of high-dose tamoxifen were administered to 8-week-old trigenic SLC34a1GFPCreERT2 (SLC34a1GCE), Rosa26-tdTomato (R26tdTomato), and *db/db*. Trigenic mice with SLC34a1GCE, R26tdTomato, and *db/m* were used as non-diabetic controls. (B) Hyperglycemia was improved by dapagliflozin. (C) Blood pressure was not different among experimental groups. (D) Increased urinary albumin excretion in *db/db* mice was improved by dapagliflozin. (E) Representative PAS staining of kidney sections and glomerulus. (F) Dapagliflozin reduced the glomerular size in *db/db* mice. N = 4–5 mice in each group. For all groups, data are means ± SEM, Bar = 50 μm in (E).

After injection of tamoxifen at 8 weeks 5 times, the mice (SLC34a1GCE-R26tdTomato-*db/db* or *db/m*) were used for experiments (Figure 1A). Three mouse groups were included in this experiment: *db/m* mice as non-diabetic controls, and *db/db* mice treated with 1.5 mg/kg/day of dapagliflozin (*db/db*-dapa) or vehicle (*db/db*) by drinking water for 8 weeks (Figure 1A). Blood glucose levels in *db/db* mice were significantly higher than those in *db/m* and *db/db*-dapa mice (Figure 1B). In contrast to previous findings on the antihypertensive effects of SGLT2i, no significant difference was noted in systolic BP between *db/db* mice and *db/db*-dapa (Figure 1C). Amount of urinary albumin excretion was larger in *db/db* mice, and it was ameliorated by dapagliflozin administration (Figure 1D). Regarding renal histology, on PAS staining of kidney sections, no evident tubular injury was observed in all experimental groups. Glomeruli were enlarged in *db/db* mice, whereas those in *db/db*-dapa mice were slightly smaller (Figure 1E and 1F).

**Figure 2:**
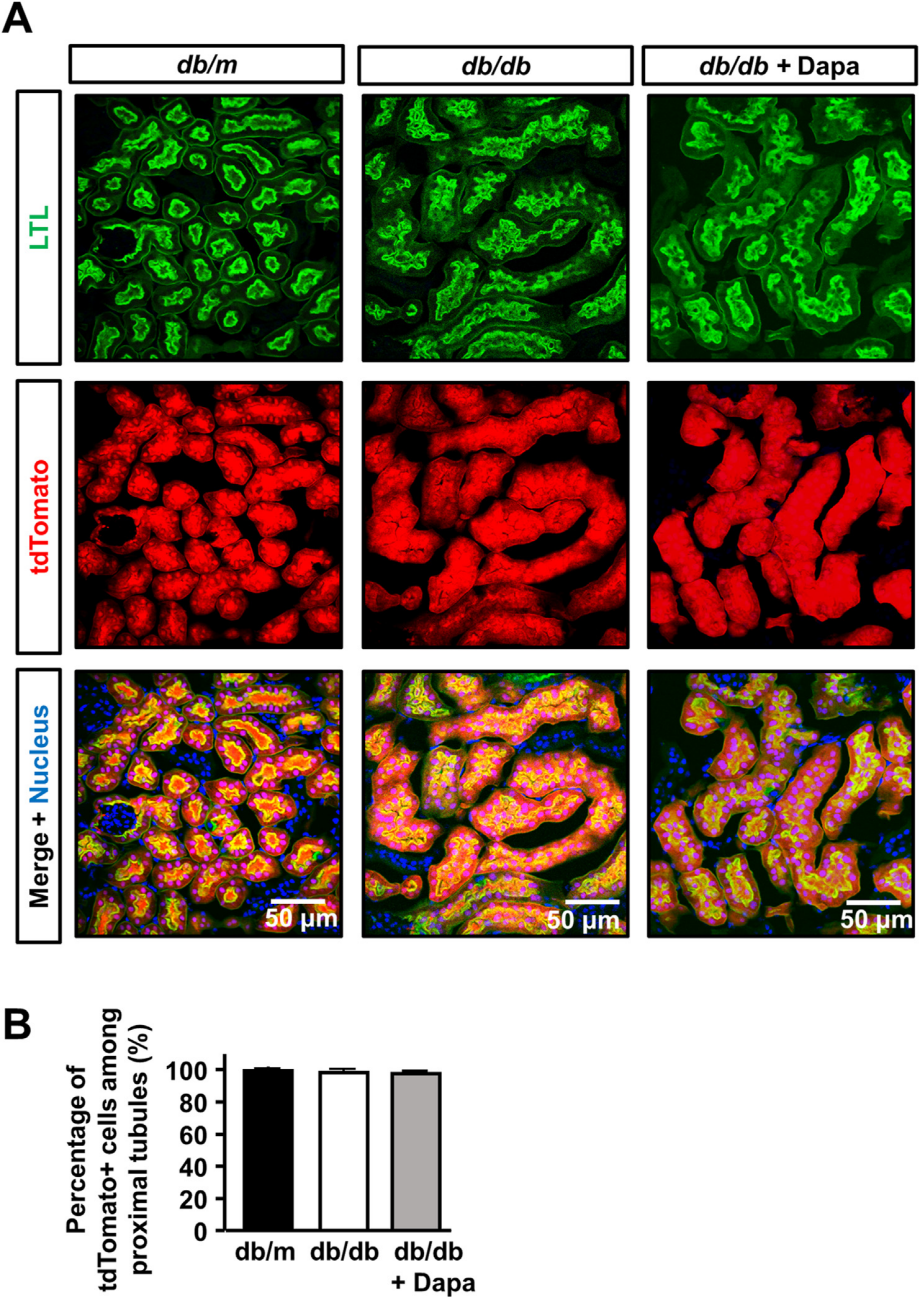
**Analysis of recombination efficiency in type 2 diabetic mice having a proximal tubule-specific tdTomato reporter.** (A) Immunofluorescence images for LTL, tdTomato, and DRAQ5 in the kidney of *db/m*, *db/db,* and *db/db*-dapa mice. (B) Quantification of tdTomato positivity among LTL + proximal tubular epithelia. For all groups, data are means ± SEM, Bar = 50 μm in (A).

In order to exclude the possibility that long-term diabetic condition or dapagliflozin treatment affected the Cre-mediated recombination, we next evaluated the labeling efficiency in experimental groups. At the end of the experimental period, the percentages of labeled cells among proximal tubular epithelia were similar in both *db/m*, *db/db,* and *db/db*-dapa mice. This demonstrated that there was no involvement of cells from outside of tubules in diabetic kidney progression (Figure 2A and 2B).

### 3.2 Distinct transcriptional patterns and KEGG pathway analysis of gene expression in sorted proximal tubular epithelia

**Figure 3:**
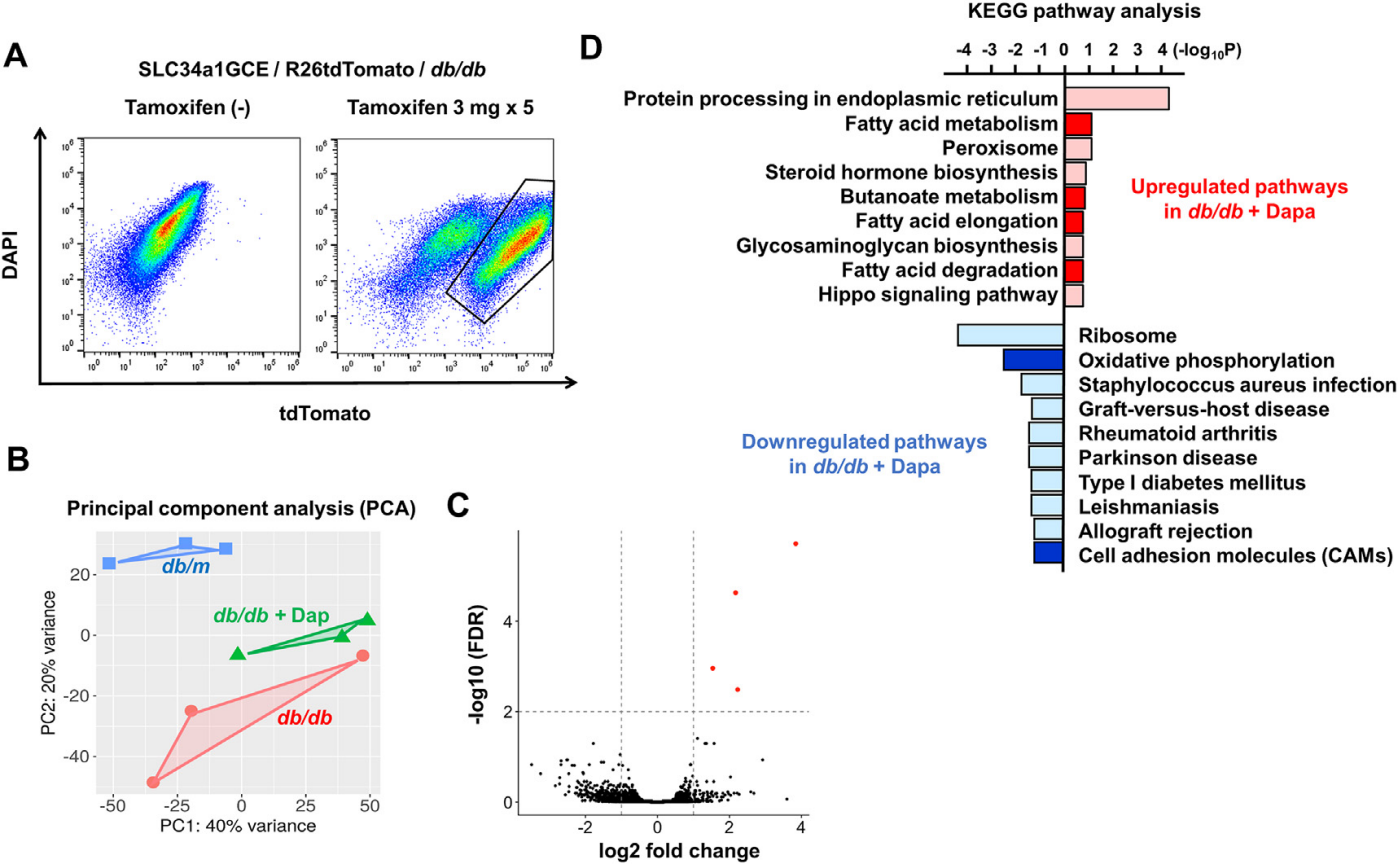
**Proximal tubule-specific transcriptomics in type 2 diabetic mice.** (A) Isolation of tdTomato + tubular epithelial cells using FACS (B) Principal component analysis (PCA) clustering with the samples from *db/m*, *db/db,* and *db/db*-dapa mouse kidneys demonstrated clear divergence among experimental groups. (C) A volcano plot showed that only 13 genes exhibited significant differential expression (p value < 0.1, absolute fold change >2) when comparing the tubular epithelia from *db/db* and *db/db*-dapa mice. Differentially expressed genes were individually color coded. (D) Enrichment analysis of the KEGG pathways for differentially expressed genes between *db/db* and *db/db*-dapa. Major significantly enriched KEGG pathways (adjusted p values < 0.05) are presented. For each KEGG pathway, the bar shows the fold-enrichment.

In order to investigate the change in proximal tubular epithelia-specific molecular pathways by dapagliflozin treatment in diabetic mice, we collected the tdTomato + tubular epithelia by FACS and RNA was extracted (Figure 3A). We further examined the differences in the transcriptome of proximal tubular epithelia between *db/db* mice treated with and without dapagliflozin by RNA sequencing. Unsupervised analysis of gene expression by the principal component analysis (PCA) revealed that the transcriptional landscapes of the *db/m*, *db/db,* and *db/db*-dapa were separated into independent clusters, indicating distinct gene expression patterns for the 3 experimental groups (Figure 3B). However, by comparing *db/db* and *db/db*-dapa, there were 13 genes among 15610 that exhibited differential expression (false detection rate-corrected p-value < 0.1, absolute fold change >2) (Figure 3C).

We annotated the gene expression using the KEGG pathway, and identified a total of 20 significantly enriched pathways (threshold p-value < 0.05) (Figure 3D). Of note, among upregulated metabolic pathways, fatty acid metabolism and butanoate metabolism were slightly upregulated in *db/db*-dapa, consistent with a previous report demonstrating the promotion of ketogenesis by SGLT2 inhibitors [18]. Among downregulated pathways, oxidative phosphorylation was downregulated more in *db/db*-dapa than in *db/db*.

### 3.3 Dapagliflozin improved kidney tissue oxygen tension and ATP content

**Figure 4:**
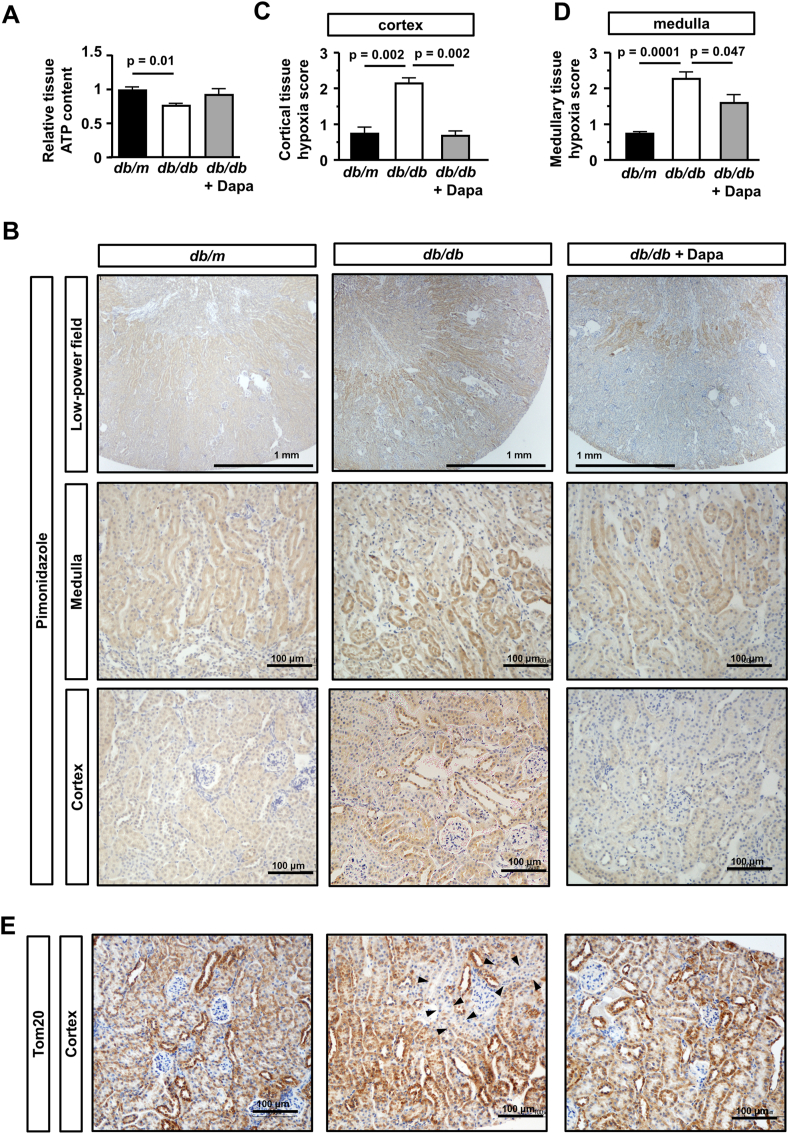
**Improvement tissue hypoxia by dapagliflozin in the kidney of type 2 diabetic mice.** (A) Relative ATP content in kidney tissue was significantly reduced in *db/db*. (B) Positive staining for pimonidazole was detected in the kidney tissue of *db/db* mice and it was ameliorated in that of *db/db*-dapa. (C) Semiquantitative analysis of pimonidazole positivity in cortical and medullary kidney tissues. Note that definitions of hypoxic score are different in the cortex and medulla. (E) Positive staining for Tom20 was observed in the kidney tissue of all experimental groups. However, in the kidney tissue of *db/db* mice, small number of Tom20 negative (arrowheads) tubules were found. Data are the means ± SEM, Bar = 1 mm in low-power images and 100 μm in high-power images in (B) and (E). n = 5 mice in each group.

As oxidative phosphorylation was downregulated in *db/db*-dapa, we measured kidney tissue ATP content. Contrary to the results of RNA sequencing, the amount of tissue ATP content was slightly higher in *db/db*-dapa than in *db/db* (Figure 4A). We further hypothesized that oxygen consumption is reduced by dapagliflozin and performed pimonidazole immunostaining to detect tissue hypoxia. In *db/m* mice, the pimonidazole-positive area was limited to within the cortico-medullary junction at which oxygen tension is the lowest under physiological conditions due to the countercurrent exchange properties of the vasa recta. In *db/db* mice, the pimonidazole-positive area patchily expanded towards the outer cortex, whereas it was rarely detected in the cortex of *db/db*-dapa (Figure 4B and 4C, Supplementary figure 1). In contrast, the pimonidazole-positive area in the medulla was similar in *db/db* and *db/db*-dapa (Figure 4B and 4D), suggesting that the effects of dapagliflozin on tissue oxygenation are more prominent in the cortex in which SGLT2 expression is predominantly expressed. We lastly examined the mitochondrial integrity by immunostaining of Tom20, a protein of mitochondrial membrane. Positive staining of Tom20 was observed in the tubules of all experimental groups (Figure 4E, Supplementary figure 2). However, patchy defect of positive Tom20 staining within the tubules was found only in *db/db* mice (Supplementary figure 2), suggesting that local tissue hypoxia might influence the mitochondrial integrity.

## 4 Discussion

This study addressed the proximal tubule-specific cellular responses in dapagliflozin-treated type 2 diabetic mice by RNA seq of FACS sorted cells from newly generated mice having a proximal tubule-specific fluorescent reporter. Pathway analysis revealed that dapagliflozin downregulated the related gene expression of oxidative phosphorylation in proximal tubular epithelial cells. Together with reduced staining of pimonidazole in the renal cortex and higher renal tissue ATP content in dapagliflozin-treated mice, dapagliflozin reduced oxygen consumption and ATP production in proximal tubules of diabetic kidneys.

Many molecular mechanisms are involved in the renoprotective effects of SGLT2 inhibitors, and improvement of renal tissue hypoxia by SGLT2 inhibitors has emerged as a strong candidate [19, 20]. Clinical and experimental investigations suggesting that SGLT2 inhibitors ameliorate renal tissue oxygen tension, especially within the cortex [7, 21, 22], support this concept. Renal tubular epithelial cells, especially proximal tubular cells, function in the reabsorption of molecules from filtered urine, which requires a massive amount of ATP produced through oxidative phosphorylation even under normal conditions [8]. In the diabetic kidney, increased glucose and sodium reabsorption through SGLTs results in the higher activity of Na-K ATPase and oxygen consumption [23]. The non-selective SGLT inhibitor phlorizin ameliorated the oxygen consumption and Na-K ATPase activity in a type 1 diabetic rodent model [23]. Consistent with previous observations, the ATP content decreased in the kidney tissue of *db/db* mice despite the upregulation of oxidative phosphorylation.

Renal tissue hypoxia is commonly found in chronic kidney disease and is considered a predictor of the rapid deterioration of renal function [24]. Several modalities for detecting tissue hypoxia have been applied [25], and pimonidazole staining was used in this study. Pimonidazole is a 2-nitroimidazole compound that accumulates in hypoxic cells via covalent binding with macromolecules or by forming reductive metabolites after reduction of its nitro group at oxygen levels below 10 mmHg [26]. Positive staining of pimonidazole is found in the outer strip of the outer medullary region in the kidney even under normal conditions [27, 28, 29]. In the diabetic kidney, several previous reports, including ours, demonstrated that the pimonidazole-positive area expands towards the cortex, suggesting that proximal tubules are exposed to a more hypoxic environment [7, 30, 31]. However, recent reports using direct measurement of intravascular oxygen tension demonstrated that SGLT2 inhibition does not affect the microvascular oxygen tension in the superficial cortex, which proposed the concept of improvement of tissue hypoxia in the renal cortex by SGLT2 inhibitors [32]. The discrepancy among these findings may be explained by the following: First, pimonidazole does not reflect the microvascular oxygen tension faithfully because this probe is used for detecting the low intracellular oxygen concentration [25]. After delivery from capillaries, oxygen diffuses from the cell membrane to inside the cell. Even if the intravascular oxygen tension is maintained, the intracellular oxygen concentration may further decrease in the case of higher oxygen demands such as in proximal tubular epithelia in diabetic kidneys. Second, the hypoxic region was diffusely observed in the outer stripe of the outer medulla in the diabetic kidney, whereas the hypoxic region in cortex was patchy. This suggests that renal hemodynamics and oxygen utilization in the renal cortex were not uniform under diabetic conditions, which affects the distribution of the hypoxic area in the cortex of the diabetic kidney.

Although blood glucose was significantly reduced in dapagliflozin-treated mice, RNA-seq demonstrated fewer differentially expressed genes between dapagliflozin-treated and -untreated mice, which is a potential limitation of this study. One possible reason is that we administered dapagliflozin through the drinking water. Compared with oral gavage, the drug concentration is maintained at lower level, which can affect gene expression. Second, gene expression is sometimes affected during the FACS procedure, including the mechanical and enzymatic tissue digestion, which may mask small differences in gene expression.

In conclusion, our proximal tubule-specific transcriptomics-based analysis demonstrated the efficacy of dapagliflozin in maintaining renal oxygen tension in type 2 diabetic mice. This may play a role in the renoprotective effects of SGLT2 inhibitor observed in large-scale clinical trials. However, whether these effects are found in advanced DKD is unknown and future experimental studies are warranted.

## Declarations

### Author contribution statement

Noriko Uehara-Watanabe; Tetsuro Kusaba: Conceived and designed the experiments; Performed the experiments; Analyzed and interpreted the data; Contributed reagents, materials, analysis tools or data; Wrote the paper.

Natsuko Okuno-Ozeki; Itaru Nakamura; Tomohiro Nakata; Kunihiro Nakai; Tomoharu Ida; Noriyuki Yamashita; Michitsugu Kamezaki; Yuhei Kirita; Satoaki Matoba; Keiichi Tamagaki: Analyzed and interpreted the data.

Aya Yagi-Tomita: Conceived and designed the experiments; Performed the experiments; Analyzed and interpreted the data.

### Funding statement

The study was funded by an investigator-initiated grant from 10.13039/100004325AstraZeneca and 10.13039/501100013170Ono Pharmaceutical. T. Kusaba reports receiving commercial research support from 10.13039/501100013170Ono Pharmaceutical, 10.13039/100004325AstraZeneca, and Taisho Pharmaceutical holdings, and speaker’s bureau honoraria from 10.13039/100004325AstraZeneca.

### Data availability statement

Data associated with this study has been deposited at Gene Expression Omnibus under the accession number GSE185801.

### Declaration of interests statement

The authors declare no conflict of interest.

### Additional information

No additional information is available for this paper.

## Appendix A Supplementary data

20220726 DMN Dapa RNAseq suppl 20220726 DMN Dapa RNAseq suppl
