# Supplementary material for: Proximal tubular epithelia-specific transcriptomics of diabetic mice treated with dapagliflozin
Source: Heliyon. 2022 Sep 13;8(9):e10615. doi: 10.1016/j.heliyon.2022.e10615 (PMC9485043; doi:10.1016/j.heliyon.2022.e10615)
Supplement: 20220726 DMN Dapa RNAseq suppl [file mmc1.docx]

**Supplementary Information**

**Proximal tubular epithelia-specific transcriptomics of diabetic mice treated with dapagliflozin**

Noriko Uehara-Watanabe, Natsuko Okuno-Ozeki, Itaru Nakamura, Tomohiro Nakata, Kunihiro Nakai, Aya Yagi-Tomita, Tomoharu Ida, Noriyuki Yamashita, Michitsugu Kamezaki, Yuhei Kirita, Satoaki Matoba, Keiichi Tamagaki, and Tetsuro Kusaba

**1 supplementary figure**


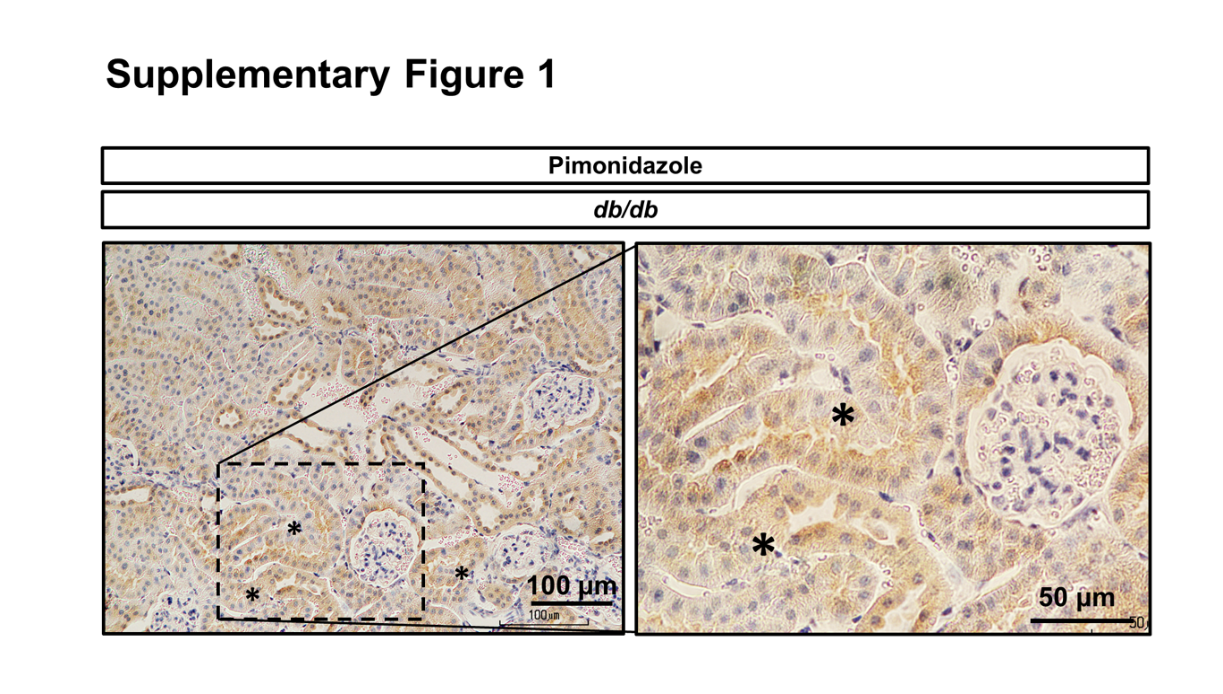


**Supplementary Figure 1. Tissue hypoxia in the kidney of type 2 diabetic mice**

Positive staining for pimonidazole was patchily detected in the tubules including convoluted proximal tubule (*) of *db/db* mice.

**
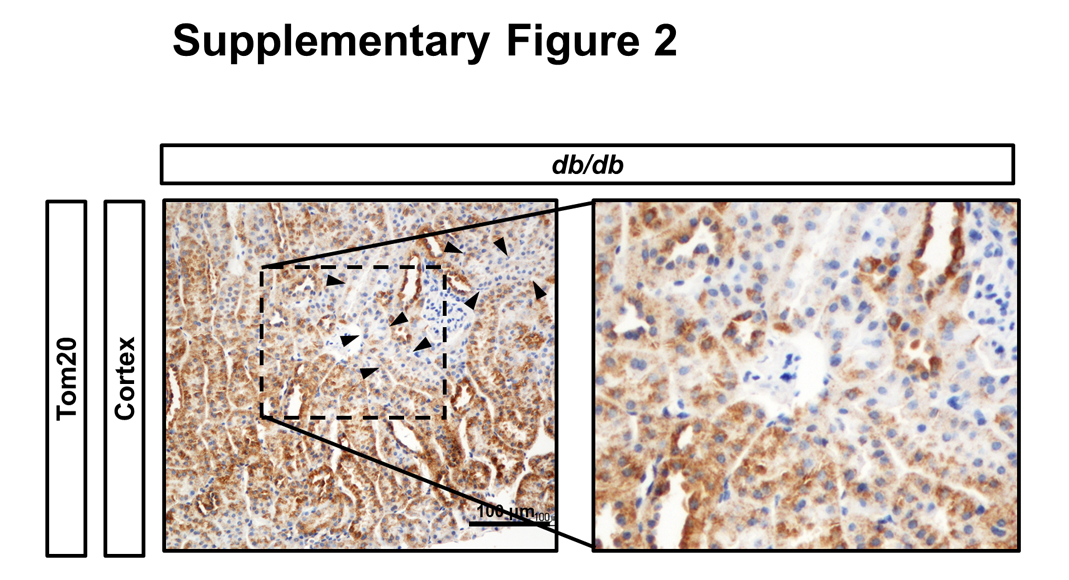
**

**Supplementary Figure 2. Detailed Tom20 staining in the kidney of type 2 diabetic mice**

High power field pictures of Tom20 staining. Reduced positive staining for Tom20 was patchily detected in the tubules of *db/db* mice. Bar = 50 μm.
